# The complex kinetics of blood endocan during the time course of sepsis and acute respiratory distress syndrome

**DOI:** 10.1186/s13054-019-2383-z

**Published:** 2019-03-12

**Authors:** Alexandre Gaudet, Erika Parmentier, Sylvain Dubucquoi, Julien Poissy, Thibault Duburcq, Lucie Portier, Philippe Lassalle, Nathalie De Freitas Caires, Daniel Mathieu

**Affiliations:** 1 Univ. Lille, U1019 – UMR 8204 – CIIL – Center for Infection and Immunity of Lille, F-59000 Lille, France; 2 CNRS, UMR 8204, F-59000 Lille, France; 3 INSERM, U1019, F-59000 Lille, France; 4 CHU Lille, Pôle de Réanimation, Hôpital Roger Salengro, F-59000 Lille, France; 5 CHU Lille, Institut d’Immunologie, Centre de Biologie Pathologie Génétique, F-59000 Lille, France; 6 Lunginnov, 1 rue du Pr Calmette, F-59000 Lille, France; 7 Institut Pasteur de Lille, F-59000 Lille, France

Dear Editor,

Several works have explored the blood concentrations of endocan in sepsis and acute respiratory distress syndrome (ARDS). However, data from the literature seem apparently conflicting, with high endocan levels being associated with either good or poor prognosis according to the different studies. Indeed, endocan levels on intensive care unit (ICU) admission correlate with the severity of sepsis [1]. In septic shock patients without ARDS at admission, high levels of endocan are found predominantly in patients who do not develop ARDS [2]. Ioakeimidou et al. reported that progression to ARDS in septic patients was associated with the increase of blood endocan during follow-up [3]. Furthermore, Orbegozo et al. and Tsangaris et al. reported that higher endocan levels measured at the clinical onset of ARDS were associated with poor respiratory outcomes [4, 5]. The above-stated observations suggest that endocan’s predictive values may sound more complex than a simple association between high plasmatic levels and the development of poor outcomes.

To better understand the evolution of endocan over the time course of sepsis and ARDS, we conducted a post hoc analysis of the kinetics of blood endocan over 72 h, based on the data from a previously published cohort of 72 septic patients without ARDS on baseline [2]. Among the 72 patients enrolled in this cohort, 11 subjects developed an ARDS at 72 h (8 mild, 3 moderate, 1 severe).

In patients without ARDS, endocan continually decreased during the 72-h time course following enrollment, with median [IQR] values falling from 9.2 [5.6–14.8] ng/mL on enrolment to 3.9 [2.6–7.7] ng/mL 72 h later (Fig. 1a). In patients progressing to mild ARDS, endocan moderately increased from 2.5 [1.3–3.4] ng/mL on enrolment to 4.1 [2.3–7.3] ng/mL at 72 h (Fig. 1b). We observed a higher increase of blood endocan in patients progressing to moderate and severe ARDS, with median [IQR] values rising from 4.7 [2.5–5.4] ng/mL on enrollment to 11 [9.5–12.6] ng/mL at 72 h (Fig. 1c).

**Fig. 1:**
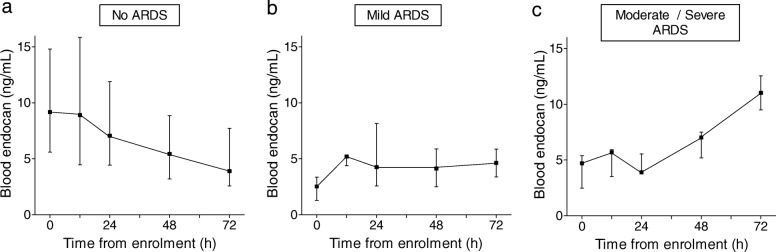
Kinetics of endocan in severe sepsis according to the presence and severity of ARDS at 72 h. Diagnosis and severity of ARDS was assessed in accordance with the Berlin definition on baseline, and at 12 h, 24 h, 48 h, and 72 h following enrollment. For each patient, the severity of ARDS corresponds to the worst level of severity reached during follow-up. Median [IQR] values of blood endocan are represented at each time point of follow-up in patients with no ARDS (**a**), mild ARDS (**b**), and moderate to severe ARDS (**c**) at 72 h. Variation of blood endocan over 72 h was significantly different between these three subgroups (Kruskal-Wallis test, *p* < 10^−2^

This study highlights the kinetics of endocan in severe sepsis and ARDS, thus helping to understand the apparently conflicting results observed in the literature. However, the interpretability of this work remains limited given the small effectives in each subgroup of ARDS, yet it may be used jointly with other data from the literature to elaborate a model of endocan’s kinetics during severe sepsis and ARDS. Therefore, further explorations are required to comfort these results.

## Abbreviations

ARDS: Acute respiratory distress syndrome
ICU: Intensive care unit
